# Painful Lumbosacral Plexopathy: A Case Report

**DOI:** 10.1097/MD.0000000000000766

**Published:** 2015-05-01

**Authors:** Edvard Ehler, Oldřich Vyšata, Radek Včelák, Ladislav Pazdera

**Affiliations:** From the Department of Neurology (EE), Regional Hospital and Faculty of Health Studies, University of Pardubice; Department of Neurology (OV), University Hospital Hradec Králové; Department of Radiology (RV), Regional Hospital Pardubice; and Department of Neurology (LP), Health Centre Rychnov nad Kneznou, Czech Republic.

## Abstract

Patients frequently suffer from lumbosacral plexus disorder. When conducting a neurological examination, it is essential to assess the extent of muscle paresis, sensory disorder distribution, pain occurrence, and blocked spine. An electromyography (EMG) can confirm axonal lesions and their severity and extent, root affliction (including dorsal branches), and disorders of motor and sensory fiber conduction. Imaging examination, particularly gadolinium magnetic resonance imaging (MRI) examination, ensues. Cerebrospinal fluid examination is of diagnostic importance with radiculopathy, neuroinfections, and for evidence of immunoglobulin synthesis. Differential diagnostics of lumbosacral plexopathy (LSP) include metabolic, oncological, inflammatory, ischemic, and autoimmune disorders.

In the presented case study, a 64-year-old man developed an acute onset of painful LSP with a specific EMG finding, MRI showing evidence of plexus affliction but not in the proximal part of the roots. Painful plexopathy presented itself with severe muscle paresis in the femoral nerve and the obturator nerve innervation areas, and gradual remission occurred after 3 months. Autoimmune origin of painful LSP is presumed.

We describe a rare case of patient with painful lumbar plexopathy, with EMG findings of axonal type, we suppose of autoimmune etiology.

## INTRODUCTION

In lumbar plexus disorders, symptoms appear in various extents of the lower torso, pelvis, and legs. Lumbosacral plexopathy (LSP) occurs relatively frequently. It represents a serious diagnostic challenge because of the extent of affliction and determining the cause, as well as differential diagnostics.^1^ LSP manifests prominent pain in some patients only. This raises the issue of the therapy, pain control, and also the treatment of the underlying causes.

## CASE REPORT

A previously healthy 64-year-old man developed, at night, 3 weeks after a light upper respiratory tract infection, severe pains in the area of the left thigh, buttock, and pelvis. Within a week, weakness of the left leg occurred, with buckling of leg, recurvation of the knee, and unstable pelvis. The patient also presented sensory disorders with unpleasant dysesthesia in thigh, buttock, and the area of the left medial tibia area. The patient was not able to walk and when lying down he had to lift and move his thigh using his hand. No sphincter dysfunctions or blocked lower spine were detected. Pains occurred predominantly in the hypogastric region, inguinal region, and the front of the thigh. The patient lost 10 kg of weight.

Clinical findings showed lower reflexes at L2–4 level in the left leg and brisk on the right, L5-S2 were 0 in the left leg and + in the right leg, cremasteric and anal reflexes were brisk bilaterally, sensory disorder from L2 in the left leg, perianogenital sensation unaltered, flexion weakened (2/5), as well as adduction (2/5), thigh abduction less weakened (3/5), lower leg extension (2/5), peripheries without paresis, and worse pelvis fixation when standing on the left leg. The left thigh circumference was 3 cm smaller, and the patient also presented small atrophy of left buttock and lower leg. The patient was able to walk several small steps using an elbow crutch (Tables 1 and 2).

**TABLE 1:**
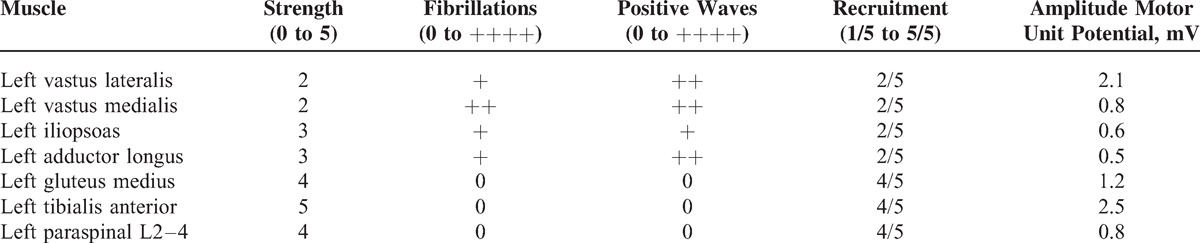
Needle Electromyography Data

**TABLE 2:**
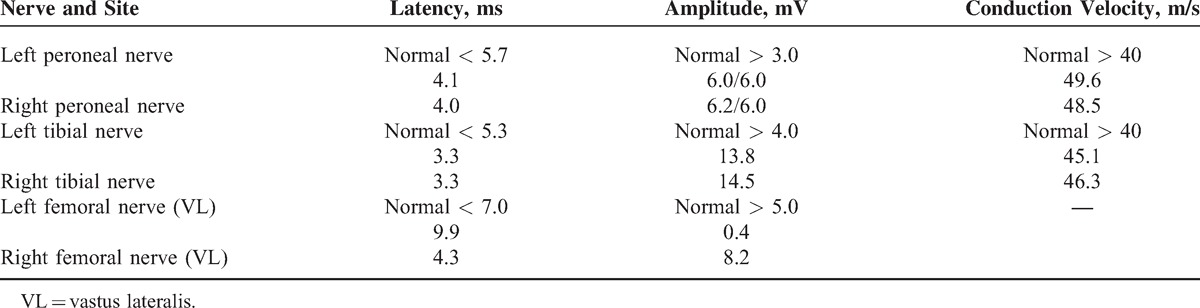
Motor Nerve Conduction Studies

A motor nerve neurography showed a left femoral nerve disorder (DML 9.8 ms, compound muscle action potential 0.4 mV). A needle electromyography (EMG) confirmed a partial denervation syndrome (with fibrillations and positive waves, as well as motor unit potential changes) in the iliacus, rectus femoris, vastus lateralis, adductor longus, and gluteus minimus muscles on the left. No abnormalities were found in the tibialis anterior muscle and paraspinal muscles on the left (L2–4), and in the rectus femoris on the right.

The cerebrospinal fluid (CSF) findings were within limits of a broader norm (protein 0.49, glucose 3.7, chlorides 128, monocytes 5/3) and the laboratory samples findings were within the norm (a glucose tolerance was within the limits). No neuroinfections were detected either in serum or in CSF. Autoimmune disease testing and oncological screening were also negative.

**FIGURE 1:**
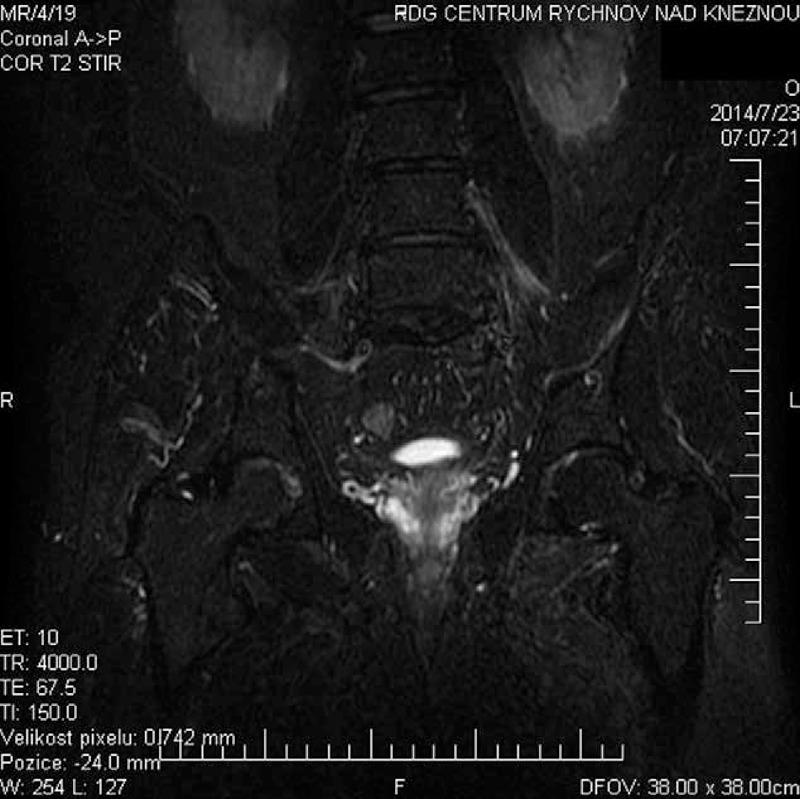
Coronal shrot tau inversion recovery: edema of left lumbar plexus with femoral nerve swelling. Muscle swelling in the region of hip joint is only marginally visible.

**FIGURE 2:**
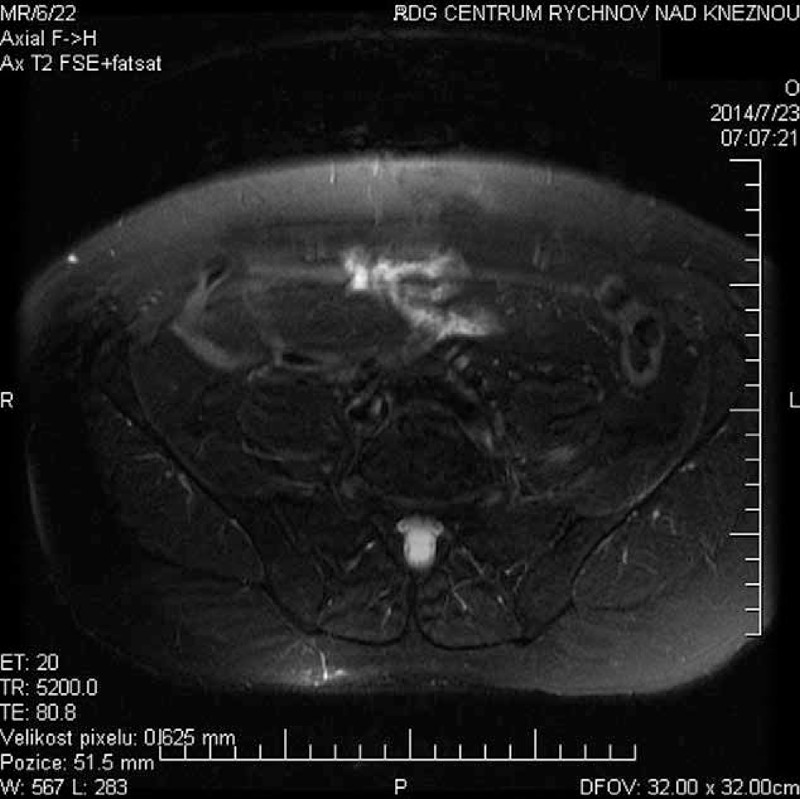
Axial T2 weighted—fat saturation: edema of left lumbar plexus.

**FIGURE 3:**
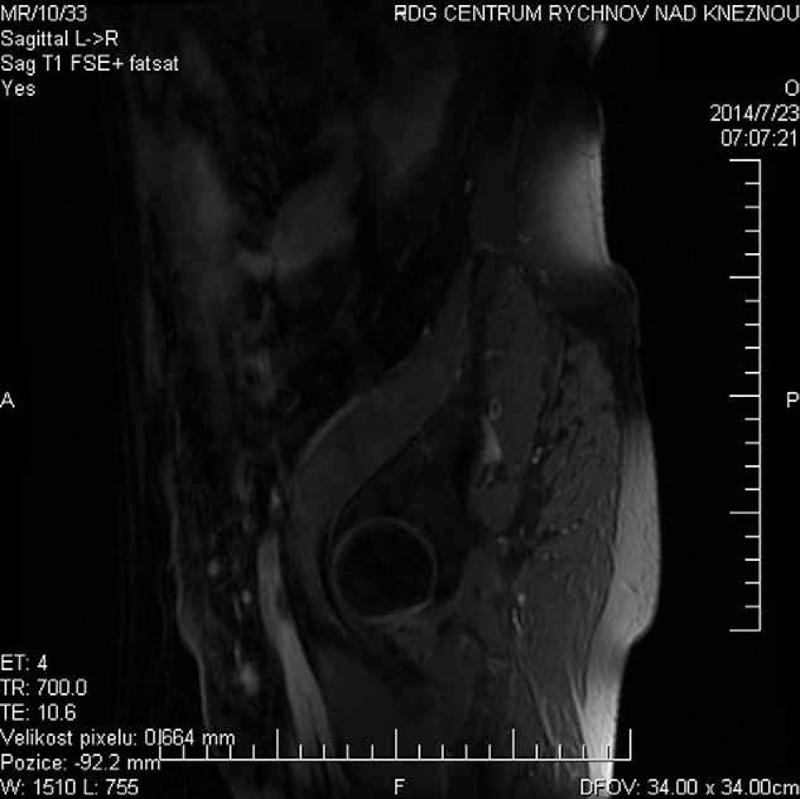
Sagittal T1 wieghted—fat saturation after gadolinium: increased enhancement of edematous left femoral nerve in the course of iliopsoas muscle.

A gadolinium magnetic resonance imaging (MRI) showed an edema with a higher enhancement of the visible course of the femoral nerve, paravertebrally from its roots until the level of the inguinal ligament. A slight edema of muscle structures—pectineus muscle, obturatorius externus, quadratus femoris, adductor brevis, adductor longus, and, peripherally, also adductor magnus on the left—is shown in Figures 1–3.

The patient was given symptomatic treatment; analgesics were prescribed, including opioids and gabapentin, for neuropathic pain relief. Rehabilitation was carried out regularly. After 3 months, gradual alleviation of pain as well as a partial recovery of strength of both the thigh and the buttock occurred.

However, after 4 months, the patient developed right thigh and buttock pain, quickly accompanied by hip and knee weakness. The patient manifested moderate sensory disorders and lumbar block was missing. The patient was prescribed 3 g of methylprednisolone intravenously (for 3 days), and then 60 mg of prednisone orally for a period of 1 week. The pain disappeared and substantial regression of the paresis occurred.

## DISCUSSION

Plexopathy diagnosis determination is conditioned by confirmation of lumbosacral plexus lesion and ruling out affliction of nerve roots or cauda within degenerative or other lumbar disease. Root affliction is evidenced by distribution of paresis and disorders, as well as neurophysiological findings (including confirmed denervation syndrome in paraspinal muscles).^2^ A needle EMG is capable of confirming localization and extent of the axonal lesion; however, the patient has to be examined only after 3 to 4 weeks from the onset of pain.^3^ Fibrillation and even positive waves are also often detected in muscles that are clinically very little afflicted.^4^ In unilateral affliction, it is necessary to compare neurophysiological findings with the healthy side.^5^ A missing prominent spinal block and missing pain provocation when the spine is moved provide an argument against radicular compression etiology. Spinal cord affliction would manifest also symptoms of disorder of long neural pathways or “sphincter dysfunctions.” Pathologic investigations are primarily from distal nerve biopsies (sural) and show multifocal axonal loss and ischemic changes due to inflammation in microvessels. There is evidence of upregulation of inflammatory mediators.^3^

A patient with a peripheral motor neuron disease (ALS) has a different distribution of paresis, is without pain and sensory disorders, and has a good SNAP recalling. Our very painful patient did not show either lumbar block or traction maneuvers. Disease progress was characterized by fast emerging paresis with pains followed by gradual pain alleviation and subsequently by regression of both paresis and sensory disorders. Denervation syndrome in paraspinal muscles was not confirmed. Paresis development was confined to innervation areas of individual nerves. An MRI confirmed plexus affliction occurring only after leaving the spinal canal.

Patients suffering from lumbar plexus plexopathy are referred to EMG laboratory quite often. Predominantly, they are type 2 diabetes patients with weight loss and (both anterior and posterior) thigh and buttock pain. This proximal diabetic neuropathy afflicts 0.8% of all diabetics^6^. The second most numerous group with lumbar plexus plexopathy is of cancer patients. This concerns gastrointestinal tumors (colon or rectal cancer), gynecological cancer, renal cancer, prostate cancer, and lymphoma.^7^ This might involve pressure on lumbosacral plexus (eg, ovarian cancer), proliferation through the plexus or perineural growth (which is characteristic of some tumors—eg, prostate cancer), postsurgical plexus lesion, paraneoplastic syndrome, and radiation-induced plexopathy.^8^ These late plexus lesions related to radiation manifest with latency period of up to several years more often occur without pain; when examined neurophysiologically, they manifest fibrillations, positive waves, motor units loss, and myokymia, which are characteristic for postradiation plexopathy.^9^

Radiculopathy was ruled out by EMG findings (missing spontaneous activity in paraspinal muscles), as well as by CSF findings and MRI, wherein inflammatory changes with edema occurred only after leaving the spinal canal. Chronic inflammatory demyelinating polyneuropathy, which would, in our patient with pain, sensory disorders, and paresis, be normally suspected on the basis of differential diagnosis, was ruled out by EMG findings (motor nerve neurography without conduction velocity reduction, without signal desynchronization, or blocks), CSF findings (no prominent hyperproteinorachy), MRI (roots were not afflicted), and, finally, clinical findings. (Most findings corresponded to lumbar plexus disorder and significant absence of distal nerve affliction, and findings were unilateral only.)

Painful LSP quite often occurs with certain neuroinfections (herpes simplex and varicella zoster).^10^ It is one of the most common neural symptoms of Lyme borreliosis, in the form of meningoradiculitis (Bannwarth syndrome). In this form, the roots are afflicted already in the spinal canal. It is clinically manifested as meningeal irritation, painful radiculopathy (with both lumbar and thoracic localization). Paresis is usually severe. After alleviation of pain, gradual regression of paresis occurs. There is occurrence of mononuclear pleocytosis and high blood protein, sometimes only with γ-globulins. A spontaneous activity (fibrillation) is noted in the paraspinal muscles. The basic diagnostic method is detection of antibodies in serum and CSF (first immunoglobulin M enzyme-linked immunosorbent assay testing and then polymerase chain reaction).

Plexopathy in our patient began suddenly at night, with pain and, shortly, with paresis and muscle atrophy; the patient lost weight. No neuroinfections, metabolic, or cancer causes were confirmed. Because of the course of the disease and EMG and MRI findings, autoimmune etiology was determined. Painful LSP with demonstrated axonal type is analogous to “acute brachial plexus neuritis” (Parsonage–Turner syndrome). Certain case studies describe painful lumbosacral temporally related to vaccination—DTP (diphtheria, tetanus, pertussis), oral polio vaccine, and *Haemophilus influenzae* vaccine.^11^

Painful idiopathic LSP afflicts lumbar plexus predominantly, although sacral plexopathy or complete LSP might also occur, albeit rarely. It is a monophasic disease, whereas relapses and continuous progression are exceptional. Unpleasant dysesthesia with burning feet occurs with relative frequency and two thirds of patients suffering from idiopathic LSP have impaired glucose tolerance.^12^

Having ruled out a surgical cause of plexopathy (tumor or vascular), the therapy is mostly symptomatic. In painful forms, it is necessary to apply nonsteroidal anti-inflammatory drug in combination with opioids and drugs against neuropathic pain. In the acute stage of idiopathic inflammatory LSP, corticotherapy is most often recommended (methylprednisolone 3 g intravenously). A certain success was also reached in corticoids applied orally, plasmapheresis, and intravenous immunoglobulins. When strictly assessed, none of the mentioned ways of treatment influencing immune response has a statistically proven effect.

## CONCLUSION

We describe a rare case of patient with painful lumbar plexopathy, with EMG findings of axonal type, we suppose of autoimmune etiology. MRI proved edema of femoral (obturator) nerve with edematous painful muscles but without radicular changes in the dural sac.^1^ This uncommon painful LSP resemble the painful brachial plexopathy.^3,5^

## References

[1] Planner AC Donaghy M Moore NR Causes of lumbosacral plexopathy. *Clin Radiol* 2006; 61:987–995. 17097418 10.1016/j.crad.2006.04.018

[2] Barr K Electrodiagnosis of lumbar radiculopathy. *Phys Med Rehabil Clin Am* 2013; 24:79–91. 10.1016/j.pmr.2012.08.011 23177032

[3] Pourmand R Immune-Mediated Neuromuscular Diseases. Basel: Karger; 2009.

[4] Laughlin RS Dyck PJB Electrodiagnostic testing in lumbosacral plexopathies. *Phys Med Rehab Clin N Am* 2013; 24:93–105. 10.1016/j.pmr.2012.08.014 23177033

[5] Wilbourn AJ Plexopathies. *Neurol Clin* 2007; 25:139–171. 17324724 10.1016/j.ncl.2006.11.005

[6] Bhanushali MJ Muley SA Diabetic and non-diabetic lumbosacral radiculoplexus neuropathy. *Neurol India* 2008; 56:420–425. 19127036 10.4103/0028-3886.44814

[7] Oberndorfer S Grisold W Lumbosakrale plexopathien bei tumorpatienten. *J Neurol Neurochir Psychiatr* 2004; 5:14–16.

[8] Hébert-Blouin MN Amrami KK Robert PM Adenocarcinoma of the prostate involving the lumbosacral plexus: MRI evidence to support direct neural spread. *Acta Neurochir* 2010; 152:1567–1576. 20473531 10.1007/s00701-010-0682-x

[9] Pradat PF Delanian S Said G Krarup C Late radiation injury to peripheral nerves. *Handbook of Clinical Neurology, Vol. 115*. Amsterdam, The Netherlands: Elsevier; 2013 743–758. 10.1016/B978-0-444-52902-2.00043-6 23931813

[10] Van Alfen N Malessy MJA Said G Krarup C Diagnosis of brachial and lumbosacral plexus lesion. *Handbook of Clinical Neurology, Vol. 115*. Amsterdam, The Netherlands: Elsevier; 2013 293–310. 10.1016/B978-0-444-52902-2.00018-7 23931788

[11] Marin R Bryant PR Eng GD Lumbosacral plexopathy temporally related to vaccination. *Clin Pediatr* 1994; 35:175–177. 10.1177/000992289403300312 8194297

[12] Amato AA Russell JA Neuromuscular Disorders. 2008; New York: McGraw Hill Medical, 20.

